# Atypical Presentation of Bordetella pertussis in a Six-Month-Old with Cleaved Lymphocytes and Mild Respiratory Distress

**DOI:** 10.7759/cureus.85395

**Published:** 2025-06-05

**Authors:** Ali Z Ansari, Zayn I Haque, Nicholas A Mokodanski, Srihita Patibandla, Sanim Choudhury, Dallas J Petroff, Sahar Hafeez

**Affiliations:** 1 Department of Pathology and Laboratory Medicine, William Carey University College of Osteopathic Medicine, Hattiesburg, USA; 2 Department of Pediatrics, Merit Health Wesley, Hattiesburg, USA; 3 Department of Internal Medicine, University of South Florida, Tampa, USA; 4 Department of Family Medicine, Ross University School of Medicine, Bridgetown, BRB; 5 Department of Internal Medicine, Trinity Health Grand Rapids, Grand Rapids, USA; 6 Department of Internal Medicine, Idaho College of Osteopathic Medicine, Boise, USA; 7 Department of Ophthalmology, Idaho College of Osteopathic Medicine, Boise, USA

**Keywords:** antibiotic treatment, atypical presentation, bordetella pertussis, cleaved lymphocytes, infant respiratory infection, lymphocytosis, pcr testing, pediatric blood smear, vaccine-escaped strains, whooping cough

## Abstract

*Bordetella pertussis*, the causative agent of whooping cough, typically presents in infants with paroxysmal coughing, inspiratory whoop, and post-tussive vomiting; however, atypical presentations are increasingly recognized, particularly in partially or recently immunized individuals. We present the case of a six-month-old previously healthy, fully immunized female infant who was evaluated for a three-day history of low-grade fever, feeding difficulties, increased irritability, and mild respiratory symptoms, in the absence of the characteristic pertussis cough or respiratory distress. Physical examination was largely unremarkable, with no signs of cyanosis, retractions, or abnormal lung sounds. Laboratory testing revealed leukocytosis with marked lymphocytosis, while a peripheral blood smear showed the unexpected presence of cleaved lymphocytes, a finding typically associated with viral infections or lymphoproliferative disorders rather than with pertussis. Despite the non-classical clinical and hematologic features, *B*. *pertussis* was confirmed by polymerase chain reaction (PCR) testing of a nasopharyngeal swab. The patient was treated with azithromycin and supportive care, resulting in clinical improvement and resolution of hematologic abnormalities. This case highlights the diagnostic challenges of pertussis in infants presenting without hallmark symptoms and introduces cleaved lymphocytes as a potential, though uncommon, hematologic feature of the infection.

## Introduction

*Bordetella pertussis* is a Gram-negative, coccobacillus bacterium that causes pertussis, commonly referred to as whooping cough. Pertussis is a highly contagious respiratory disease that primarily affects infants, young children, and adults with waning immunity [1]. Despite the global success of vaccination programs, which have significantly reduced the burden of pertussis, the disease remains a serious public health issue [2]. This resurgence of pertussis has been attributed to multiple factors, including a decrease in vaccine efficacy over time, vaccine hesitancy, and incomplete vaccine coverage in certain populations [3]. The shift from whole-cell to acellular vaccines, while associated with fewer side effects, has led to concerns about their relatively shorter duration of immunity, particularly among adolescents and adults who may act as reservoirs for transmission [4]. Current acellular pertussis vaccines are thought to confer shorter-lived immunity and provide limited mucosal protection, which may facilitate asymptomatic carriage and transmission. Moreover, recent genetic changes in circulating *B*. *pertussis* strains have been identified as contributing to the persistence of the disease despite vaccination efforts [5].

Pertussis remains a leading cause of vaccine-preventable deaths in young children globally, with infants who have not yet completed their primary vaccination series or who are too young to be fully vaccinated being particularly vulnerable [2]. Due to their immature immune systems, infants are at risk for more severe and prolonged illness compared to older children or adults. Classic pertussis in this age group typically presents in three stages: catarrhal, paroxysmal, and convalescent. The catarrhal stage begins with mild symptoms such as rhinorrhea, sneezing, low-grade fever, and a mild cough, resembling a common cold. The paroxysmal stage is marked by sudden and severe coughing fits that can lead to the characteristic “whooping” sound on inspiration, post-tussive vomiting, and in some cases, apnea and cyanosis. The convalescent phase involves a gradual reduction in symptoms, with coughing episodes decreasing in frequency and severity over several weeks [6]. However, not all cases follow this classic trajectory, and infants may exhibit atypical manifestations, making diagnosis challenging and often delayed. In vaccinated infants, the absence of the hallmark cough or “whoop” complicates the clinical picture, as mild, nonspecific symptoms such as irritability, poor feeding, or low-grade fever may dominate the presentation.

Laboratory findings in pertussis often include leukocytosis with absolute lymphocytosis, believed to result from pertussis toxin-mediated inhibition of lymphocyte migration [7]. This is a well-recognized feature, particularly in infants, who tend to mount a more robust hematologic response. However, in rare instances, peripheral blood smears may reveal atypical lymphocyte morphology. In this case, the identification of cleaved or lobulated lymphocytes, typically seen in viral infections such as Epstein-Barr virus (EBV) or cytomegalovirus (CMV), or in hematologic malignancies, was an unexpected and diagnostically significant finding [8]. Such morphologic changes have been infrequently reported in association with *B. pertussis* infection and may complicate clinical interpretation. The diagnosis of pertussis is confirmed through polymerase chain reaction (PCR) testing of nasopharyngeal specimens, which remains the most sensitive and specific method, particularly when performed early in the illness. This case draws attention to a potentially underrecognized hematologic manifestation of pertussis and reinforces the importance of considering pertussis in the differential diagnosis, even when laboratory features deviate from classical expectations.

## Case presentation

A six-month-old female infant, born full-term via spontaneous vaginal delivery without perinatal complications, presented to the emergency department with a three-day history of low-grade fever, mild respiratory symptoms, increased irritability, and reduced oral intake. She had no history of prior hospitalizations, chronic medical conditions, or developmental delays. Her growth parameters were appropriate for age, and she had met all developmental milestones to date. Immunizations were up to date in accordance with the national immunization schedule, including three doses of the diphtheria, tetanus, and acellular pertussis (DTaP) vaccine. There were no known drug allergies, and family history was negative for hereditary, immunologic, or respiratory conditions.

Her mother reported that the infant was feeding normally until three days prior to presentation, at which point she became increasingly fussy and began nursing less frequently and for shorter durations. On the second day, the infant was noted to be more irritable and less responsive to visual and auditory stimuli, raising concern for evolving systemic illness. By the third day, she developed a low-grade fever of 38.0°C (100.4°F), prompting medical evaluation. Throughout the illness, there were no episodes of coughing, paroxysms, whooping, apnea, cyanosis, or post-tussive vomiting. The mother denied any recent travel, sick contacts within the household, or direct exposure to known respiratory infections. However, she recalled that several infants at a local playgroup the previous week had exhibited mild upper respiratory symptoms, raising the possibility of community-acquired exposure. The infant had no vomiting, diarrhea, or signs of dehydration beyond the reported reduced feeding.

Upon arrival at the emergency department, the infant appeared mildly ill but alert and hemodynamically stable. She was irritable and exhibited decreased spontaneous activity, though she remained responsive to handling. Her vital signs at triage were as follows: a temperature of 38.1°C (100.6°F), heart rate of 130 beats per minute, respiratory rate of 40 breaths per minute, and oxygen saturation of 98% on room air. These values were within age-appropriate limits, aside from a mild fever. The respiratory examination revealed no signs of distress: there was no nasal flaring, subcostal retractions, grunting, or use of accessory muscles. Pulmonary auscultation demonstrated clear and equal breath sounds bilaterally, without wheezes, crackles, or rhonchi. There was no stridor or upper airway congestion. Cardiovascular examination revealed a regular heart rate and rhythm, with normal S1 and S2 heart sounds and no murmurs, rubs, or gallops; capillary refill was brisk, and peripheral pulses were symmetric. The abdomen was soft, non-tender, and non-distended, with normal bowel sounds and no evidence of hepatosplenomegaly. Neurologic assessment showed appropriate tone and responsiveness without focal deficits, and the infant’s fontanelle was soft and flat. The skin examination was unremarkable, with no rash, petechiae, or lesions, and no cervical or generalized lymphadenopathy was present.

Laboratory evaluation was initiated to assess for infectious and hematologic causes of the infant’s nonspecific symptoms. While culture was not performed, it is important to note that, despite its limited sensitivity and longer turnaround time in acute settings, culture remains the gold standard for epidemiologic surveillance and antimicrobial resistance monitoring. A complete blood count revealed a white blood cell count of 14,000 cells/μL (reference: 6,000-17,000 cells/μL), with a notably elevated absolute lymphocyte count of 8,000 cells/μL (reference: 4,000-7,000 cells/μL for infants), consistent with lymphocytosis. Hemoglobin, hematocrit, and platelet counts were within normal limits. Inflammatory markers, including C-reactive protein (CRP) and erythrocyte sedimentation rate (ESR), were both within normal ranges at 2 mg/L and 1 mm/hr, respectively. Liver function tests, including aspartate transaminase (38 U/L), alanine transaminase (17 U/L), and alkaline phosphatase (214 U/L), and renal function markers such as blood urea nitrogen (11 mg/dL) and creatinine (0.2 mg/dL), were all within age-appropriate reference intervals. A summary of the laboratory results is presented in Table 1.

**Table 1:** The patient’s key hematologic, inflammatory, hepatic, and renal parameters on admission. Notable findings include lymphocytosis with normal inflammatory markers and unremarkable organ function tests.

Test	Patient value	Reference range
White blood cell count	14,000 cells/μL	6,000–17,000 cells/μL
Absolute lymphocyte count	8,000 cells/μL	4,000–7,000 cells/μL (infants)
Hemoglobin	11.7 g/dL	10.5–13.5 g/dL
Hematocrit	35%	33–39%
Platelet count	260,000 cells/μL	150,000–400,000 cells/μL
C-reactive protein	2 mg/L	<10 mg/L
Erythrocyte sedimentation rate	1 mm/hr	0–10 mm/hr (infants)
Aspartate transaminase	38 U/L	20–60 U/L
Alanine transaminase	17 U/L	5–45 U/L
Alkaline phosphatase	214 U/L	150–420 U/L
Total bilirubin	0.4 mg/dL	0.1 – 1.0 mg/dL
Blood urea nitrogen	11 mg/dL	5–18 mg/dL
Creatinine	0.2 mg/dL	0.2–0.4 mg/dL (infants)

**Figure 1:**
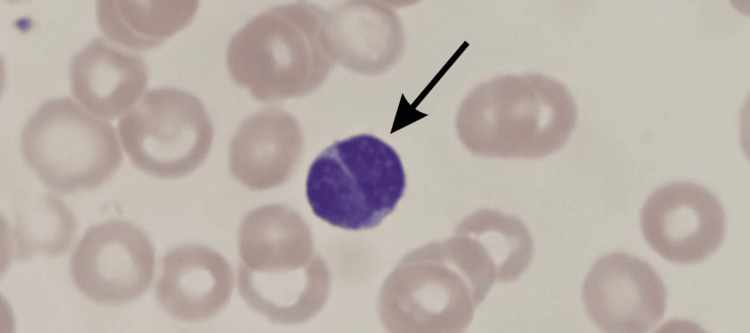
Wright-Giemsa-stained peripheral blood smear from the patient showing a cleaved lymphocyte (black arrow) with an irregular, indented, and lobulated nucleus.

A peripheral blood smear was performed and revealed a notable presence of cleaved lymphocytes, which are mature lymphoid cells with irregular, indented, or lobulated nuclei (Figure 1). These morphological features are typically associated with viral infections such as EBV or CMV, or with hematologic malignancies, making their appearance in this case both clinically unusual and diagnostically significant. Based on these findings and the nonspecific presentation, the differential diagnosis was expanded to include viral and bacterial infections as well as hematologic disorders. The initial considerations included viral upper respiratory tract infection, bronchiolitis, early-onset sepsis, and viral syndromes such as EBV or CMV. Upper respiratory tract infection and bronchiolitis were plausible due to mild fever and recent exposure, but were ultimately deemed unlikely given the absence of rhinorrhea, cough, wheezing, abnormal auscultation findings, or respiratory distress. Sepsis was also considered due to the infant’s irritability and decreased feeding; however, this was deprioritized in light of her hemodynamic stability, normal inflammatory markers, and lack of neutrophilic predominance on the complete blood count.

Further diagnostic testing included a multiplex real-time PCR panel targeting respiratory viruses, along with targeted bacterial PCR from a nasopharyngeal swab that detected the IS481 insertion sequence, acknowledging its potential cross-reactivity with *B. holmesii* and *B. bronchiseptica*, which can lead to false-positive results. A chest radiograph was not performed, as the infant showed no clinical signs of respiratory distress, hypoxia, or abnormal auscultation findings that would justify imaging at the time. The infant was admitted for supportive care and ongoing evaluation. Oxygen saturation was continuously monitored via pulse oximetry. Supplemental oxygen was initiated via nasal cannula at 1 liter per minute to maintain saturation above 95%, although the infant remained on room air for the majority of her hospital stay. Intravenous fluids were administered at a maintenance rate of 80 mL/kg/day to ensure hydration, as oral intake remained suboptimal. Antipyretic therapy with acetaminophen (15 mg/kg/dose every 6 hours as needed) was initiated to manage fever and discomfort.

The infant remained clinically stable during the initial 48 hours of admission. Intermittent low-grade fevers persisted but never exceeded 38.3°C (100.9°F). No new respiratory signs or symptoms emerged. On hospital day two, approximately seven days after symptom onset, the nasopharyngeal swab returned positive for *B. pertussis* by PCR. Despite the atypical clinical features, such as absence of paroxysmal cough, cyanotic episodes, and post-tussive emesis, the diagnosis of pertussis was confirmed. Azithromycin was initiated at a dose of 10 mg/kg on day 1, followed by 5 mg/kg/day on days 2-5, in accordance with current pediatric guidelines for the treatment of pertussis in infants under one year of age. The antimicrobial regimen aimed to reduce disease transmission, mitigate the severity of illness, and prevent secondary complications; close contacts, including household members, were also advised to receive prophylactic azithromycin, particularly to protect vulnerable groups such as neonates and pregnant women.

By hospital day five, the infant’s clinical condition had significantly improved. She exhibited reduced irritability, increased alertness, and resumed regular breastfeeding sessions. Her temperature normalized, and repeat peripheral smear on day three showed a reduction in the number of cleaved lymphocytes, indicating a resolving immune response. No additional complications were observed during hospitalization. The patient was discharged home in stable condition with instructions to complete the remaining oral doses of azithromycin and continue symptomatic care as needed. Parents were educated regarding the atypical nature of pertussis presentation in vaccinated infants, the potential for delayed onset of classical symptoms, and the importance of reducing contact with vulnerable individuals. A follow-up visit was scheduled within one week to assess recovery, ensure compliance with therapy, and monitor for the emergence of any delayed symptoms associated with pertussis.

## Discussion

Our patient, a six-month-old female infant, presented with nonspecific features including low-grade fever, increased irritability, decreased oral intake, and lethargy, without cough or respiratory distress. This clinical picture could easily be attributed to a variety of benign viral illnesses common in infancy, which initially limited the differential diagnosis. However, the infant’s laboratory evaluation provided an early clue: an elevated absolute lymphocyte count. Lymphocytosis is a well-known hematologic hallmark of pertussis, often preceding the development of the paroxysmal phase [7]. The mechanism involves pertussis toxin-mediated inhibition of lymphocyte migration from the bloodstream into lymphoid tissues, resulting in elevated circulating lymphocytes [9]. Importantly, lymphocyte predominance on the differential, though not pathognomonic, may serve as an early clue to pertussis, particularly in the absence of classical respiratory symptoms. In this case, the total leukocyte count was only mildly elevated, and lymphocytes comprised 57% of the differential, a pattern that can also be observed in viral infections. While this finding raised suspicion, pertussis-specific testing such as loop-mediated isothermal amplification (LAMP) was not initially performed; instead, the diagnosis was ultimately established through a multiplex PCR panel, which offered broader coverage of potential respiratory pathogens.

An unusual and diagnostically notable feature of this case was the identification of cleaved lymphocytes on peripheral blood smear-mature lymphoid cells with irregular, indented, or lobulated nuclei. While these morphologic changes are more typically associated with viral infections such as EBV or CMV, or hematologic malignancies like lymphoblastic lymphoma, their unexpected presence in a confirmed case of pertussis broadened, rather than narrowed, the initial diagnostic considerations [8]. This prompted targeted viral serologies and molecular testing to rule out other etiologies. Although cleaved lymphocytes are not specific to pertussis, their detection in this context emphasizes the potential for atypical hematologic responses in bacterial infections and highlights how such findings can influence the scope and direction of early diagnostic evaluation, particularly when clinical signs are subtle or nonspecific [10].

Although the patient had received all age-appropriate DTaP immunizations, breakthrough infections can occur. The acellular pertussis vaccine, while effective in reducing disease severity and limiting transmission, does not provide complete immunity, especially in the first year of life when the primary series has not yet been completed [4]. In such cases, the disease may present in a modified or attenuated form, potentially obscuring clinical recognition. In our patient, the milder course of illness, with preserved oxygenation, absence of respiratory distress, and prompt recovery, may be attributed in part to the protective effects of prior immunization. However, the infection still posed risks, both for potential progression and for transmission to others, particularly vulnerable or unvaccinated individuals. While we initially described this case as an atypical presentation, it is worth considering whether the observed features more accurately reflect a vaccine-modified clinical course rather than true atypia. Mild or non-classical symptoms are increasingly recognized in partially or fully immunized individuals, particularly in infants who have not completed the full DTaP series. From this perspective, our case may represent a common manifestation of pertussis in the context of partial immunity rather than a clinical outlier. Clarifying this distinction is essential for improving diagnostic awareness and informing public health strategies.

The diagnosis of pertussis in this case was ultimately confirmed by nasopharyngeal PCR testing, which has emerged as the diagnostic modality of choice for *B. pertussis* due to its rapid turnaround time, high sensitivity, and excellent specificity. PCR is particularly valuable during the early stages of illness when bacterial load is highest and cultures, though historically considered the gold standard, are less likely to yield positive results due to their lower sensitivity, longer incubation times, and technical limitations [11]. In clinical scenarios where the presentation is atypical or subtle, as in this infant, PCR plays a critical role in supporting a diagnosis that might otherwise be missed. The utility of this test is especially evident in young infants who may not manifest hallmark respiratory symptoms, and in whom early detection is essential to guide appropriate management. In this case, early identification through molecular testing facilitated the prompt initiation of macrolide antibiotic therapy, specifically azithromycin, and allowed for timely implementation of infection control precautions, thereby reducing the risk of transmission to other vulnerable individuals, including household contacts and community members.

Treatment with azithromycin, dosed appropriately for the patient’s weight and age, was initiated in accordance with current Centers for Disease Control and Prevention (CDC) guidelines for the management of pertussis in infants [12]. Macrolide antibiotics, particularly azithromycin, are the first-line agents due to their proven efficacy in eradicating *B. pertussis* from the respiratory tract. In this case, the infant demonstrated a favorable clinical response, with gradual resolution of her systemic symptoms, improved feeding behavior, and normalization of hematologic parameters, including a decline in cleaved lymphocytes.

This case highlights several important considerations for clinicians. First, pertussis should remain on the differential diagnosis in young infants presenting with nonspecific symptoms such as fever, irritability, or feeding difficulties, particularly when lymphocytosis is present. Second, laboratory findings that deviate from typical expectations, such as the presence of cleaved lymphocytes, should prompt a broadened diagnostic approach but also be interpreted within the full clinical context. Third, it reinforces the limitations of current acellular pertussis vaccines in providing sterilizing immunity and the need for heightened awareness of breakthrough infections.

## Conclusions

This case illustrates the diagnostic complexity of *B. pertussis* infection in infants, particularly when the clinical presentation lacks hallmark features such as paroxysmal cough, whooping, or respiratory distress. Although the infant had received age-appropriate DTaP immunizations, breakthrough infection occurred, and the clinical course was milder than classic descriptions. Rather than representing a truly atypical case, the presentation may reflect a vaccine-modified form of pertussis, an increasingly recognized phenomenon in partially or fully immunized individuals. The presence of cleaved lymphocytes on peripheral smear, though uncommon in pertussis, served as a valuable diagnostic clue that broadened the differential diagnosis and prompted further testing. This highlights the critical role of thorough clinical assessment and attention to hematologic findings in identifying pertussis in its less overt forms. Early diagnosis via PCR enabled prompt antibiotic therapy, which contributed to the patient’s favorable outcome and reduced the risk of transmission. Clinicians should remain alert to pertussis in young infants, even among those who are vaccinated and lack classical symptoms. Greater awareness of vaccine-modified presentations, along with careful use of diagnostic tools, is essential to ensure timely recognition and appropriate management of pertussis in this vulnerable population.
